# Relationship Between Group Work Competencies and Satisfaction With Project-Based Learning Among University Students

**DOI:** 10.3389/fpsyg.2022.811864

**Published:** 2022-02-09

**Authors:** Anabel Melguizo-Garín, Iván Ruiz-Rodríguez, María Angeles Peláez-Fernández, Javier Salas-Rodríguez, Elena R. Serrano-Ibáñez

**Affiliations:** ^1^Department of Social Psychology, Social Work, Social Anthropology, and East Asian Studies, University of Málaga, Málaga, Spain; ^2^Department of Personality, Assessment, and Psychological Treatment, University of Málaga, Málaga, Spain

**Keywords:** group work, competencies, project-based learning, higher education, satisfaction

## Abstract

There is a growing interest in improving the teaching–learning process at all levels of education, including higher education. In recent years, university institutions have been taking action to renew and modernize the way in which they teach and learn, making the process more dynamic and closer to the current social reality. Competencies such as the ability to work in a team have become essential for the successful implementation of innovative methodologies in which student participation is particularly relevant. Student acceptance is key to the success of any teaching methodology; however, the influence of group work skills on satisfaction with innovative methodologies such as project-based learning (PBL) has not yet been tested among university students. Thus, the objective of this study is to explore the association between group work competencies and satisfaction with PBL. A total sample of 359 students from two Spanish universities participated in the research. Our results reveal that there is a significant and positive relationship between competencies related to group work and satisfaction with PBL. In addition, a multiple regression analysis shows that the competencies “Conception of group work,” “Usefulness of group work,” “Planning of group work by teachers,” and “Group norms” increase satisfaction with the use of the PBL methodology. This work expands our knowledge about the role in increasing students’ satisfaction that is played by the ability of college students to work as a team. These findings could also guide teachers interested in new teaching methodologies.

## Introduction

In recent decades, we have been subjected to accelerated change due to new technologies, evolving toward the Information and Knowledge Society. This aspect has also had to be integrated into higher education and its teaching–learning processes. Students start from an abundance of information that is accessible and attractive. However, they lack the skills to manage this information and to know how to use this knowledge to solve day-to-day problems. In this sense, higher education must go beyond the knowledge taught, and teach skills. This aspect has been highlighted by the United Nations Educational, Scientific and Cultural Organization (UNESCO) (1999), which establishes, as the objectives of education, “to be,” “to know,” “to do,” and “to work together.”

To respond to this need, higher education has developed new student-centered teaching–learning methodologies such as project-based learning (PBL; Barrett, 2010). PBL is a type of dynamic methodology that is very suitable for improving the teaching–learning processes of students (Chen et al., 2020). PBL is based on collaborative work between people in a group who must self-direct their work to meet an objective (Clark, 2006). Specifically, PBL presents real problems to students, who must solve them by reflecting and investigating. To do this, they must cooperate, integrate the knowledge of each member of the group, and work both alone and as a team (Wiek et al., 2011).

The implementation of PBL in the higher education system is currently gaining special relevance, as it contributes to the development of work competencies that are very appropriate for the challenges currently faced by society (Kolmos, 1996; Kolmos et al., 2021). There have been studies in higher education that have successfully applied PBL in scientific disciplines of different types, such as chemistry (Morales, 2009), analytical chemistry (Belt et al., 2002), biology (Allen and Tanner, 2003; Pantoja and Covarrubias, 2013), physics (Van Kampen et al., 2004), physiology (Mierson, 1998), and earth sciences (Higgs, 2005). PBL has also been used in other areas of higher education, such as English (Kamiskiené and Januliené, 2006), education (Iglesias, 2002; Imaz, 2015; Toledo-Morales and Sánchez-García, 2018; Granado-Alcón et al., 2020), history (Galindo, 2008), psychology (López-Zafra et al., 2015; Wiggins et al., 2016), and law (Cubero-Truyo and Díaz-Ravn, 2010), where its use as a didactic strategy has led to significant improvements.

Project-based learning integrates academic knowledge with real-world practices, and undergraduates may not only acquire knowledge and skills and use these in the context of a subject, but may also work on their general competencies (e.g., critical thinking or self-awareness; Brassler and Dettmers, 2017).

In PBL, students have a very high degree of participation and involvement (Kirschner et al., 2006). Given that PBL is a dynamic, interactive, and cooperative methodology, the most relevant competencies involved in PBL are social skills (that is, social, cognitive, and emotional competences that facilitate interaction with others) and group/teamwork skills, that is a set of behaviors, attitudes, and knowledge that contribute toward the struggles of a group to achieve specific common objectives (Stout et al., 1996). There are six skills that are essential for an individual to work efficiently in a group/team: communication, adaptability, coordination, interpersonal skills, decision making, and leadership (O'Neil et al., 1999). In the implementation of participatory and innovative methodologies that require student participation, like PBL, it is necessary to focus on students’ teamwork skills and social competencies (El-Adaway et al., 2015). Sometimes PBL may not be beneficial, or its implementation may be difficult, precisely because of the high level of student participation required.

The literature has highlighted the positive aspects of this methodology in contrast to traditional teaching classes and work that is excessively directed by the teacher; these aspects include a greater development of competencies associated with scientific work, a greater knowledge transfer, and a greater satisfaction and affinity with the subject among the students.

Student acceptance is a key to the success of any teaching methodology. Although past research has tested the satisfaction of university students with subjects in which a PBL methodology was used, the influence of group work skills on students’ satisfaction with PBL has not yet been tested. In addition, most of the studies mentioned have compared a traditional lecture class to a PBL class in a single degree or at most two degrees, which might limit the generalizability of the research to other degrees.

Therefore, the objective of this study was to explore the association between group work competencies and satisfaction with PBL among a sample of students studying for several different degrees, thus contributing to the body of knowledge about PBL methodology in higher education.

## Materials and Methods

### Participants and Procedure

A total of 359 students from the University of Málaga and the University Isabel I de Castilla took part in the study. The sample consisted of students taking five degree courses: Tourism, Social Work, Psychology, Audiovisual Communication, and Labor Relations and Human Resources. A convenience sampling technique was used, since participants were enrolled in subjects taught by some of the authors of the study. The questionnaires were applied after the students had carried out a piece of group work in their degree subject using the PBL methodology. In order to increase the sincerity of the students, questionnaires were handed by an independent collaborator and students were informed that the answers to the questionnaires were anonymous. The participants completed questionnaires along with a sociodemographic data survey. Although the composition of the sample was biased in favor of females, this imbalance was in line with the gender distribution in the degree courses in which the study was carried out. In fact, female representation in the Tourism and Social Work degree courses is 68 and 85%, respectively, and these are the degree courses from which the majority of the sample came. All participants gave their consent to participate in the study.

The questionnaires were applied after the students had carried out a piece of group work in their degree subject using the PBL methodology. The participants completed questionnaires along with a sociodemographic data survey.

### Instruments

The *Questionnaire for the Analysis of Cooperation in Higher Education* (ACOES) was applied in order to measure group work competencies in these higher education students (García et al., 2012). The format response is a five-point Likert scale, ranging from 1 (“Strongly disagree”) to 5 (“Strongly agree”). The ACOES comprises 49 items grouped into seven dimensions: Conception of group work, which assesses the student’s mental representations and meaning regarding group work (five items; Cronbach *α* coefficient = .79); Usefulness of group work, aimed at evaluating student’s opinion regarding the usefulness of group work in promoting social interactions, independent learning, and future job performance (six items; *α* = .80); Planning of group work by teachers, which analyses student’s opinion concerning the quantity, complexity, coordination, and mentoring of cooperative work carried out by the teacher (four items; *α* = .73); Criteria for organizing groups, that assess the criteria that students consider relevant to form group’s work: academic o personal reasons, composition (homogeneous or heterogeneous in relation to age, sex, or experience), or temporal stability (eight items; *α* = .54); Group norms, which evaluate if group norms should be designed by the teacher, the students, or negotiated between both agents (nine items; *α* = .64); Internal functioning of groups, that assess the sequence of tasks carried out by the students before the final product (seven items; *α* = .78); and Effectiveness of group work, aimed at evaluating the external and internal conditions of the group that generate better performance and production levels (weighting of group work in the final grade, information on the criteria used, discrimination in the evaluation of the various personal contributions, and inclusion of student self-evaluation and peer evaluation; 10 items; *α* = .82). The mean scores for each dimension of the ACOES were calculated by adding the scores of the items grouped in the dimension and then dividing by the number of items comprising the dimension.

For the purpose of measuring student satisfaction with PBL, and following the guidelines in the literature on PBL assessment (Dochy et al., 2003; Peterson, 2004; Egido et al., 2007), a *Satisfaction with PBL* (SPBL) self-reporting questionnaire was designed *ad hoc*. The scale comprised 17 items with a five-point Likert scale format response from 1 (“Strongly disagree”) to 5 (“Strongly agree”). Cronbach’s coefficient was .93. Principal component analysis of the SPBL scale was carried out. Bartlett’s test of sphericity was statistically significant [*χ*^2^ (136) = 3307.97, *p* < .001], so the factor analysis was applicable to the selected items. The KMO test shows that the strength of the relationships between the items was high (KMO = .931). Since the scale was designed to assess students’ general satisfaction with PBL, the decision was made to extract a single factor. The result was a factor with an eigenvalue of 8.24 that explained 48.46% of the variance. All items had factor loadings higher than .3, which is the minimum limit for considering the factor loading to be significant (Floyd and Widaman, 1995). The mean score for the SPBL was calculated by adding the scores of the items and then dividing by the number of items comprising the questionnaire.

### Analytical Strategy

The data analysis was carried out with IBM SPSS version 23. Descriptive statistics were obtained for the socio-demographic variables. Correlational analysis was applied in order to test the association between the SPBL and the ACOES dimensions. Furthermore, a multiple regression analysis with an enter method was carried out with the purpose of checking the predictive value of the dimensions of the ACOES over the SPBL.

## Results

### Descriptive Analysis and Relationship Between Variables

Descriptive statistics of the participants are shown in Table 1. A total of 359 students (74.9% female), with an age range of 18–58, participated in the study. The average age was 22.31 years (*SD* = 4.30).

**Table 1:** Participant sociodemographic information (*N* = 359).

Age	22.31 (4.30)^a^
Gender	% (*N*)
Male	25.1 (90)
Female	74.9 (269)
**Degree**	
Labor Relations and Human Resources	19.9 (73)
Tourism	42.3 (155)
Psychology	5.2 (19)
Social Work	28.4 (104)
Audiovisual Communication	4.1 (15)

a Mean (*SD*).

The correlation coefficients are shown in Table 2. These show that satisfaction with PBL was significantly associated with all the ACOES dimensions.

**Table 2:** Correlations of Analysis of Cooperation in Higher Education (ACOES) dimensions with Satisfaction with project-based learning (PBL).

	Satisfaction with PBL
Conception of group work	.495^**^
Usefulness of group work	.556^**^
Planning of group work by the teachers	.492^**^
Criteria for organizing groups	.278^**^
Group norms	.250^**^
Internal functioning of groups	.363^**^
Effectiveness of group work	.345^**^

** *p* ≤ .01.

### Predictive Model of Satisfaction With PBL

Table 3 shows the results of the multiple regression analysis using the ACOES dimensions as predictors. The regression analysis revealed a significant model for satisfaction with PBL (*F* = 58.14, *p* < .001, *R*^2^ = .40). The conception of group work (*𝛽* = .13, *p* < .05), the usefulness of group work (*𝛽* = .32, *p* < .001), the planning of group work by teachers (*𝛽* = .26, *p* < .001), and the group norms (*𝛽* = .12, *p* < .01) were significant predictors. The other ACOES dimensions were not significant.

**Table 3:** Multiple linear regression model of the dimensions of ACOES for the satisfaction with PBL.

	Unstandardised coefficients		Standardised coefficients	*t*
	*B*	*SE*	*β*	
(model)	.844^***^	.234		3.615
Conception of group work	.125^*^	.056	.132	2.224
Usefulness of group work	.325^***^	.061	.318	5.331
Planning of group work by the teachers	.218^***^	.041	.257	5.352
Group norms	.132^**^	.047	.118	2.779

* *p* < .05;

** *p* < .01;

*** *p* < .001.

*R* = .630; *R*^2^ = .396; *R*^2^ adjusted = .390; and *F* = 58.136.

## Discussion

Project-based learning is currently gaining special relevance in the higher education system. This methodology requires collaborative work, for which several teamwork skills and social competencies of the students are relevant (El-Adaway et al., 2015). However, it has not been analyzed whether these skills could be related to satisfaction with PBL. Thus, the study aim was to explore the relationship between group skills and students’ satisfaction with PBL. The results indicate that satisfaction with the implementation of PBL is related to the teamwork skills of the students. Group competencies, such as “Conception of group work,” “Usefulness of group work,” “Planning of group work by teachers,” and “Group norms” increase satisfaction with the use of the PBL methodology. These skills are closely related to the planning and organization of group work and the role played by the teacher in supporting the planning of the work. These results are in line with previous studies that found association between student satisfaction with PBL and individual and group variables including group harmony (Kilgour et al., 2016), highlighting that the student’s own abilities to relate and structure the group can be very important for their satisfaction with the process.

The findings of this study have relevant theorethical implications as they relate group skills and student satisfaction with the PBL methodology. The relationship between both variables is a novel aspect in research of higher education, and underline the need to consider group competencies in future research on higher education employing PBL methodology. On the other hand, these findings have practical implications, as they suggest that the quality of higher education could be improved if teachers played an active role in the implementation of the PBL methodology and designed suitable plans for the group work to be carried out in the implementation of PBL. These plans should address aspects like the design of the group work, the objectives and potential usefulness of the group work, and the rules to be followed by the group, since these issues are closely related to the students’ satisfaction with this methodology. In addition, these findings can give guidance, to teachers who want to use new methodologies in their teaching, on the prior skills that are necessary if such an experience is to be satisfactory. Additionally, the inclusion in higher education training of plans aimed at enhancing teamwork skills for students and teachers could be a positive strategy, since such skills could be relevant if the experience of participatory methodologies is to be satisfactory.

A limitation of this research is its cross-sectional character; in future research, a longitudinal study with a control group will be carried out in order to discover whether training in teamwork skills improves satisfaction with PBL. The sample is limited in terms of its origin, since the students belonged only to two universities: University of Málaga and University Isabel I de Castilla, but a positive aspect is that they were studying for different degrees, which contributes to the representativeness of the sample. Another positive aspect of this research lies in its practical implications, since knowing which group competencies can improve satisfaction with PBL can be positive and useful information for improving students’ experience with this participatory methodology.

It would be interesting for future research on higher education to explore other individual an contextual factors that may influence satisfaction with PBL methodology, such as the academic performance of students, the number of students in class, or the arrangement of the spaces. In order to generalize the results, it would be appropriate to use samples of students of various university degrees and in different levels and courses. Likewise, the research could be extended to teachers, exploring whether there is a relationship between their abilities and satisfaction with the PBL methodology.

In conclusion, the findings of this research provide a novel aspect in the study of the benefits of using PBL-based methodologies in higher education, as they found that some group competencies, such as group norms, conception of group work, planning of group work by teachers, and perceived usefulness of group work enhance students’ satisfaction with PBL. These results suggest that in order to improve the quality of higher education, it would be relevant for teachers to incorporate these group competencies in the study plans and didactics of their subjects. To contribute to the successful implementation of PBL in higher education, it would be useful that both teachers and students were trained with specific group tools such as the ability to plan and organize a teamwork.

## Data Availability Statement

The original contributions presented in the study are included in the article/supplementary material, further inquiries can be directed to the corresponding authors.

## Ethics Statement

The studies involving human participants were reviewed and approved by The Research Ethics Committee of the University of Málaga. The patients/participants provided their written informed consent to participate in this study.

## Author Contributions

AM-G, IR-R, MP-F, JS-R, and ES-I wrote the first draft. AM-G created and organized the study. AM-G, MP-F, JS-R, and ES-I collected the data. IR-R created the database. IR-R and JS-R analyzed the data. All authors contributed to the article and approved the submitted version.

## Funding

This research was supported and funded in part through research projects from the University of Málaga PIE19-004.

## Conflict of Interest

The authors declare that the research was conducted in the absence of any commercial or financial relationships that could be construed as a potential conflict of interest.

## Publisher’s Note

All claims expressed in this article are solely those of the authors and do not necessarily represent those of their affiliated organizations, or those of the publisher, the editors and the reviewers. Any product that may be evaluated in this article, or claim that may be made by its manufacturer, is not guaranteed or endorsed by the publisher.
